# High dose intermittent sorafenib shows improved efficacy over conventional continuous dose in renal cell carcinoma

**DOI:** 10.1186/1479-5876-9-220

**Published:** 2011-12-21

**Authors:** Xiaoen Wang, Liang Zhang, S Nahum Goldberg, Manoj Bhasin, Victoria Brown, David C Alsop, Sabina Signoretti, James W Mier, Michael B Atkins, Rupal S Bhatt

**Affiliations:** 1 Department of Radiology, Beth Israel Deaconess Medical Center, Harvard Medical School, Boston, Massachusetts, USA; 2 Division of Hematology-Oncology and Cancer Biology, Beth Israel Deaconess Medical Center, Harvard Medical School, Boston, Massachusetts, USA; 3 Division of Interdisciplinary Medicine and Biostatistics, Beth Israel Deaconess Medical Center, Harvard Medical School, Massachusetts, USA; 4 Department of Radiology, Hadassah Hebrew University Medical Center, Jerusalem, Israel; 5 Department of Pathology, Brigham and Women's Hospital, Harvard Medical School, Boston, Massachusetts, USA

**Keywords:** Renal cell carcinoma, anti-angiogenic therapy, arterial spin labeled magnetic resonance imaging

## Abstract

**Background:**

Renal cell carcinoma (RCC) responds to agents that inhibit vascular endothelial growth factor (VEGF) pathway. Sorafenib, a multikinase inhibitor of VEGF receptor, is effective at producing tumor responses and delaying median progression free survival in patients with cytokine refractory RCC. However, resistance to therapy develops at a median of 5 months. In an effort to increase efficacy, we studied the effects of increased sorafenib dose and intermittent scheduling in a murine RCC xenograft model.

**Methods:**

Mice bearing xenografts derived from the 786-O RCC cell line were treated with sorafenib according to multiple doses and schedules: 1) Conventional dose (CD) continuous therapy; 2) high dose (HD) intermittent therapy, 3) CD intermittent therapy and 4) HD continuous therapy. Tumor diameter was measured daily. Microvessel density was assessed after 3 days to determine the early effects of therapy, and tumor perfusion was assessed serially by arterial spin labeled (ASL) MRI at day 0, 3, 7 and 10.

**Results:**

Tumors that were treated with HD sorafenib exhibited slowed tumor growth as compared to CD using either schedule. HD intermittent therapy was superior to CD continous therapy, even though the total dose of sorafenib was essentially equivalent, and not significantly different than HD continuous therapy. The tumors exposed to HD sorafenib had lower microvessel density than the untreated or the CD groups. ASL MRI showed that tumor perfusion was reduced to a greater extent with the HD sorafenib at day 3 and at all time points thereafter relative to CD therapy. Further the intermittent schedule appeared to maintain RCC sensitivity to sorafenib as determined by changes in tumor perfusion.

**Conclusions:**

A modification of the sorafenib dosing schedule involving higher dose intermittent treatment appeared to improve its efficacy in this xenograft model relative to conventional dosing. MRI perfusion imaging and histologic analysis suggest that this benefit is related to enhanced and protracted antiangiogenic activity. Thus, better understanding of dosing and schedule issues may lead to improved therapeutic effectiveness of VEGF directed therapy in RCC and possibly other tumors.

## Background

Recently, several new agents targeting the VEGF pathway have demonstrated promising activity in patients with advanced renal cell carcinoma (RCC). Sorafenib is a multi-tyrosine kinase inhibitor (mTKI) whose targets include vascular endothelial growth factor receptor-2 (VEGFR2) and its activity is thought to be based on its inhibition of this target. In a randomized, placebo-controlled phase III trial sorafenib prolonged progression free survival (PFS) from 2.8 months (placebo group) to 5.5 months [1]. Based on these and other data, sorafenib received FDA approval for treatment of patients with RCC in late 2005. This mechanism is particularly relevant in kidney cancer as opposed to other cancer types (e.g., HCC) where the inhibition of the serine-threonine kinase Raf is likely at least as important as the inhibition of VEGFR-2.

While these effects are highly significant, strategies to prolong the effects of sorafenib and other VEGFR TKI targeted agents are important as tumors typically develop resistance to therapy within 5-11 months. Efforts to enhance the efficacy of VEGFR TKIs have included dose intensification based on either tolerance [2], pharmacokinetic or pharmocodynamic markers [3-5]; the use of agents that more selectively inhibit the VEGFR (eg. Axitinib, tivozonib)[6] combination regimens, sequencing of agents and schedule alterations [7].

Patients exhibiting disease that progressed on sorafenib have been shown to respond to increased dose of sorafenib or the administration of sunitinib or the more selective VEGFR inhibitor axitinib [8,9] suggesting that the resistance to VEGFR targeted therapy could be overcome by more intensive inhibition of the target.

Sunitinib is typically administered in a 4 week on/2 week off schedule while sorafenib and pazopanib are administered continuously. This 2 week break in the approved sunitinib regimen was instituted to allow for patients to recover from toxicity of the 50 mg/day dose. It has been observed that some patients exhibit disease progression during this break only to later to have their disease symptoms controlled again with re-initiation of treatment (Atkins, M. Personal observation). This led to the development of a 37.5 mg/day continuous sunitinib regimen. However, this regimen appeared to be less active than the intermittent schedule in a preliminary phase II trial [10]. This observation was confirmed in a phase III trial which established that the lower dose continuous schedule had an inferior time to disease deterioration relative to the standard higher dose intermittent regimen [11]. Taken together these data support the notion that intermittent dosing of VEGF TKIs might enable administration of higher doses that could lead to enhanced antitumor effects including higher response rate and prolonged PFS.

Perfusion imaging with techniques such as arterial spin labelled (ASL) MRI have shown utility as surrogate markers for the effect of anti-angiogenic treatment in preclinical models. Serial ASL MRI imaging and staged tumor biopsies of established murine RCC xenografts have shown that sorafenib administration produces tumor necrosis in 3 days with associated loss of perfusion [12]. Together, these models have established that reperfusion occurs up to weeks in advance of actual tumor regrowth. These studies provide rationale for using ASL MRI to monitor the anti-angiogenic effects associated with different doses and schedules of VEGFR inhibitory therapies in xenograft models.

In the present work, we show that while continuous administration of conventional dose sorafenib exhibits antitumor activity in RCC, administration of a similar total amount of drug as an increased dose on an intermittent schedule results in improved anti-tumor effects and that this benefit appears to be related to enhanced antiangiogenic activity of the higher dose regimen.

## Methods

### Cell Culture

786-O cells (VHL deficient human renal cell carcinoma) were obtained from the American Type Culture Collection (ATCC, Manassas, VA) and were grown in RPMI 1640 medium from Cellgro. All media were supplemented with 2 mM L-glutamine, 10% fetal calf serum and 1% streptomycin (50 μg/ml) and cells were cultured at 37°C with 5% CO_2_.

### Tumor xenograft induction

For the subcutaneous xenograft tumor model female nude beige mice (Charles River Laboratories, MA), 6-8 weeks of age and 20 g average body weight, were used as per [13]. The mice were housed and maintained in laminar flow cabinets under specific pathogen-free conditions. All experiments were approved by the Institutional Animal Care and Use Committee at Beth Israel Deaconess Medical Center. To produce tumors, renal cancer cells were harvested from subconfluent cultures by a brief exposure to 0.25% trypsin and 0.02% EDTA. Trypsinization was stopped with medium containing 10% FBS, and the cells were washed once in serum-free medium and resuspended in PBS. Only suspensions consisting of single cells with greater than 90% viability were used for the injections.

To establish RCC tumor xenograft, an established human VHL deficient RCC cell line (786-O) was injected subcutaneously (1 × 10^7 ^cells) into the flanks of 6-8 week old nude/beige mice. Tumors developed in > 80 percent of the mice and were usually visible within 2 weeks of implantation and once they reached a diameter of 3-5 mm, were measured daily to ensure a consistent size at the outset of treatment. Tumor long and short axes were measured using calipers daily. Tumor volumes were calculated with the formula volume = length × width^2^/2 and followed to determine growth curves. Animals were euthanized according to IACUC guidelines and treatment was terminated as experimentally designed and described below. Prior to dissecting the tumor, the midpoint of the cranial caudal axis of the tumor was marked. This marked line matched the ASL imaging slice. The tumor was cut into three equal segments parallel to the marked line. The mid segment of the tumor was fixed in 10% formalin at room temperature for 24 hours prior to embedding in paraffin. Tumors were sectioned and stained with H&E, and immunohistochemical analysis.

### Sorafenib dosing

Sorafenib tosylate (80 mg/kg daily 6 of 7 days per week by gavage) was begun when the tumors had grown to a diameter of 12 mm [14,15]. This 80 mg/kg dose was based on a study by Chang et al. [16] in which four doses of sorafenib (15, 30, 60 and 90 mg/kg; free base equivalent) were compared in 786-O and RENCA xenograft mice. They demonstrated similarity in the 60 and 90 mg/kg groups in terms of growth delay. The 60 mg/kg free base dose, would convert to 82 mg/kg of the tosylate form that was used in this study. This dose (rounded to 80 mg/kg) was considered the maximally effective dose. It was used in our prior studies with this murine model [7] and was defined as the "conventional dose" for current study. The "high dose intermittent" regimen was 160 mg/kg administered 3 days on and 4 days off, "low dose intermittent" was 80 mg/kg administered 3 days on and 4 days off, and "high dose continuous" therapy was 160 mg/kg administered continuously. The continuous conventional dose and the high dose intermittent regimens delivered the same total dose of sorafenib over 7 days (80 mg/kg/day given 6 out of 7 days per week and 160 mg/kg/day 3 days per week).

Mice were grouped randomly into treatment with vehicle (n = 9), conventional dose continuous (n = 11), high dose intermittent (n = 11), conventional dose intermittent (n = 8), and high dose continuous (n = 4) by gavage when the tumors reached 12 mm in diameter. All animals were sacrificed and tumors were dissected ~36 days post therapy with the exception of vehicle treated mice which were sacrificed when tumors reached the mandated 20 mm sacrifice size (~22 days post treatment). In addition, we sacrificed 6 mice on day 3 after treatment with high dose (n = 3) and conventional dose (n = 3) for both the CD34 and CD31 analyses.

ASL MR imaging was performed on 6 mice prior at baseline (day 0), day 3, day 7, and day 10 post treatment with conventional dose continuous (n = 3) or high dose intermittent (n = 3).

### Immunohistochemistry

For CD34 analysis, 4 um thick sections were prepared from formalin-fixed, paraffin-embedded tumor specimens. Sections were deparaffinized, rehydrated and heated with a pressure cooker to 125°C for 30 seconds in citrate buffer for antigen retrieval. After cooling to room temperature, sections were incubated in 3% hydrogen peroxide for 5 minutes to quench endogenous peroxidase, (Dako, Carpinteria, CA). The anti-CD34 antibody (Abcam, Cambridge, MA, Cat # AB-8158) was applied at a 1:50 dilution, diluted with DaVinvi Green diluent (BioCare Inc, Cat# PD900L), to sections for 1 hour, followed by rabbit anti-rat secondary antibody for 30 minutes. Detection was performed by incubating with Dako EnVision+ System HRP labeled polymer anti-rabbit for 30 minutes, followed by DAB chromogen. Slides were scanned using the Scanscope XT (Aperio Technologies Inc., Visa, CA) and analyzed using a modified Microvessel analysis algorithm (Aperio Technologies Inc).

For CD31 analysis, 4 um thick sections were prepared from formalin-fixed, paraffin-embedded tumor specimens. Sections were deparaffinized, rehydrated and heated with a pressure cooker to 125°C for 30 seconds in citrate buffer for antigen retrieval. After cooling to room temperature, sections were incubated in 3% hydrogen peroxide for 5 minutes to quench endogenous peroxidase, (Dako, Carpinteria, CA). The anti-CD31 antibody (Abcam, Cambridge, MA, Cat #AB28364) was applied at a 1:50 dilution, diluted with Dako antibody diluent (Dako, Cat #S0809), to sections for 1 hour. Detection was performed by incubating with Dako EnVision+ System HRP labeled polymer anti-rabbit for 30 minutes, followed by DAB chromogen. Slides were scanned using the Scanscope XT (Aperio Technologies Inc., Visa, CA) and analyzed using a modified Microvessel analysis algorithm (Aperio Technologies Inc).

### Tumor perfusion imaging

Tumor perfusion imaging (ASL MRI) was performed as previously described [12]. Briefly mice were anaesthetized, and placed in the supine position on a 3 cm in diameter custom-built surface coil. Adhesive tape was used to limit movement. Images were acquired using a 3.0 T whole-body clinical MRI scanner (3T HD; GE Healthcare Technologies, Waukesha, WI). A single slice ASL image was obtained with a single-short fast spin echo sequence (SSFSE) using a background-suppressed, flow-sensitive alternating inversion-recovery strategy. Twenty-four label and control pair images were acquired and averaged for the ASL acquisition. A reference proton density image was acquired by turning off all background suppression and labelling pulses in the ASL preparation. T1 measurement was performed after ASL imaging by using the same imaging sequence at same slice location but with inversion recovery at different inversion times. The single transverse slice of ASL was carefully positioned at the center of the tumor, which was marked on the skin with a permanent marker pen for follow-up MRI studies. ASL sequence raw data were saved and transferred to the analysis workstation for image reconstruction by using custom software written within the Interactive Data Language (IDL; ITT visual Information Solutions, Boulder, CO). The ASL difference image, between average label and control images, was then converted to quantitative tumor perfusion as previously described [17].

Perfusion was calculated on a pixel-by-pixel basis, and quantitative maps were produced. The quantitative maps and the corresponding proton density reference images were then analyzed by using Image J software (Image Processing and Analysis in Java; National Institutes of Health, Bethesda, MD). To determine tumor perfusion, a region of interest was drawn freehand around the peripheral margin of the tumor by using an electronic cursor on the reference image that was then copied to the perfusion image. The mean blood flow for the tumor tissue within the region of interest was derived, and image window and level were fixed. A 16-color table was applied in 10 mL/100 g/min increments ranging from 0 to 160 mL/100 g/min, with flow values represented as varying shades of black, blue, green, yellow, red, and purple, in order of increasing perfusion.

### Statistical Analysis

All data were expressed as mean ± SEM. Statistical significance of differences among groups of sorafenib dosing response was calculated using one-way or two-way analysis of variance (ANOVA). Tukey's significant difference post hoc test was used for pairwise comparisons after ANOVA to correct for multiple testing. The groups among which P < 0.05 were considered significantly different.

## Results

### High dose therapy is more effective in slowing tumor growth than low dose therapy

**Figure 1:**
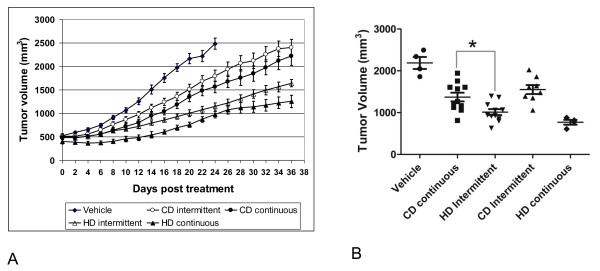
**Effects of dosing and schedule of sorafenib on tumor growth**. Treatment was initiated when tumors reached 12 mm in long axis (volume ~500 mm^3^) with the administration of vehicle or different doses and schedule of sorafenib orally as indicated. All animals were sacrificed and tumors were dissected ~36 days post treatment with the exception of vehicle treated mice which were sacrificed when tumors reached the mandated 20 mm sacrifice size (~22 days post treatment, volume ~2500 mm^3^). Figure 1A shows that treatment with high dose intermittent therapy inhibited tumor growth to a greater extent than conventional dose continuous and intermittent therapy. Data are presented as mean tumor volume with SEM. Figure 1B presents the tumor volume on the average day when vehicle treated tumors reached 20 mm in long axis (~22 days post treatment). The tumor volume of mice with high dose intermittent therapy is significantly smaller than that of conventional dose continuous therapy (P < 0.05) while the volume did not differ in the high dose intermittent vs high dose continuous or conventional dose intermittent vs conventional dose continuous arms (P > 0.05).

We treated mice bearing 786-O tumor xenografts (human VHL deficient renal cell carcinoma) on the 4 described treatment regimens: 1) Conventional dose continuous, 2) high dose intermittent therapy, 3) conventional dose intermittent therapy, and 4) high dose continuous therapy. As shown in Figure 1, treatment with high dose therapy slowed tumor growth to a greater extent than conventional dose continuous or intermittent dosing. The tumor size at the average day that the control tumors reached their terminal sacrifice size was lower in the high dose intermittent arm than in the conventional dose continuous or intermittent arms but not significantly different than the high dose continuous arm. To assess relative stability in tumor growth, the time to increase by 2 mm from pre-treatment size was calculated as this is the smallest measurable increase in tumor size and roughly correlated to the 25% increase in tumor size used in RECIST schedule for tumor progression. Table 1 shows that conventional dose continuous sorafenib stabilized tumors for 9.3 +/- 1.7 days as compared to high dose intermittent (12.9 +/- 3.3 days, P < 0.05). The high dose intermittent did not differ significantly from the high dose continuous. The conventional dose intermittent arm showed inferior anti-tumor effect with a 7.1 +/- 1.7 day time to increase by 2 mm as compared to high dose intermittent and conventional dose continuous regimens (P < 0.05). Figure 1B shows the average tumor size on the average day of sacrifice of the vehicle treated tumors. The average tumor size in the vehicle was (2188 +/- 142.6 mm^2^), in CD continuous (1372 +/- 103.7 mm^2^), HD continuous (769.1 +/- 57.82 mm^2^), CD intermittent (1552 +/- 107.3 mm^2^) and HD intermittent (1012 +/- 70.65 mm^2^). There was a significant difference in all groups including vehicle vs all treatment arms, and CD continuous vs HD intermittent (P < 0.05) except CD continuous vs CD intermittent and HD intermittent vs HD continuous.

**Table 1:** Tumor growth by 2 mm

**Treatment**	**Vehicle (N = 9)**	**CD continuous (N = 11)**	**HD intermittent (N = 11)**	**CD intermittent (N = 8)**	**HD continuous (N = 4)**
Days to grow by 2 mm	6.2 ± 1.9	9.2 ± 1.7	12.9 ± 3.3	7.1 ± 1.7	16.8 ± 3.9

High dose intermittent therapy stabilized tumors longer as seen by the difference in the number of days for the tumors to increase by 2 mm in size. All arms are significantly different (P < 0.05) except the conventional dose (CD) continuous vs CD intermittent, the CD intermittent vs vehicle and the high dose (HD) intermittent vs HD continuous.

### High dose sorafenib reduces tumor microvessel density and perfusion

**Figure 2:**
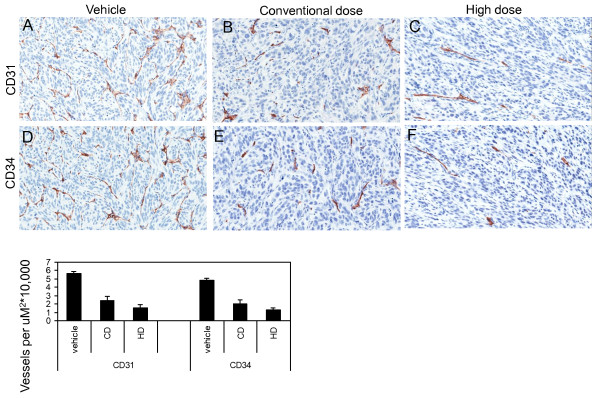
**Microvessel density analysis of sorafenib treated tumors**. Immunohistochemical analysis of CD31 (A-C) and CD34 (D-F) expression in vehicle (A, D), low dose (B, E) and high dose (C, F) at day3 are shown. The bar graph shows the average CD31 and CD34 expression in the vehicle (n = 3), conventional dose (n = 3), and high dose (n = 3) arms. As compared to vehicle treated tumors, the tumors exposed to conventional and high dose sorafenib had lower MVD (CD34 and CD31)(P < 0.01). There was a consistent trend for lower MVD in the high dose treatment as compared to the conventional dose treatment in both the CD34 and CD31 analyses.

To determine the mechanism by which the higher dose of sorafenib provided improved activity, we assessed microvessel density (MVD) of the tumors by CD34 and CD31 immunohistochemistry (IHC). Tumors were harvested after 3 days of treatment. Figure 2 shows that as compared to vehicle treated tumors, the tumors exposed to conventional and high dose sorafenib had lower MVD (CD34 and CD31)(P < 0.01). There was a consistent trend for lower MVD in the high dose treatment as compared to the conventional dose treatment in both the CD34 and CD31 analyses.

**Figure 3:**
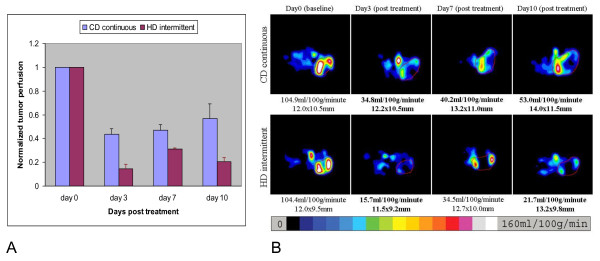
**Effect of dosing and schedule of sorafenib on tumor perfusion**. Figure 3A shows normalized serial tumor perfusion from 3 mice at 4 time points. Conventional dose of sorafenib lowers tumor perfusion by 57% by day 3. This decrease begins to resume by day 10 of therapy. In contrast, high dose intermittent sorafenib lowers perfusion by 85% at day 3. While tumor perfusion increases slightly at day 7 after there has been a 4 day lapse in treatment, by day 10, tumor perfusion is still low (20% of pretreatment). Tumor perfusion was significantly different in two arms at day 3 (P < 0.01) and day 10 (P < 0.001) as determined by two way ANOVA followed by Bonferroni posttest. Data are presented as mean normalized tumor perfusion with SEM. Figure 3B, shows a representative set of serial tumor perfusion images from mice treated with a conventional dose (upper row) or high dose intermittent (lower row) sorafenib. The blood flow values are shown in bold text are the values obtained while the mice were on sorafenib and the values that are not in bold are from mice before or off therapy. The tumor size was measured in long and short axes (mm) and the mean blood flow (mL/100 g/minute) are shown below each image. A color bar at the bottom represents the range of perfusion values from 0 to 160 mL/100 g/minute.

To understand the dynamic changes in tumor perfusion over time, we performed serial imaging with ASL MRI at day 0, 3, 7 and 10. As shown in Figure 3A, the conventional dose of sorafenib lowered tumor perfusion by 57% by day 3. Perfusion began to be restored by day 10 of therapy when the tumors were actively growing despite continued treatment with sorafenib. In contrast, high dose sorafenib lowered perfusion by 85% at day 3 (P < 0.01 for comparison to conventional dose). In the intermittent arm, tumor perfusion increased at day 7 (after a 4 day break in treatment) by 100% compared to the nadir perfusion at day 3. At day 10, 3 days after sorafenib re-administration, tumor perfusion was significantly lower in the high dose intermittent arm than in the conventional dose continuous arm (p < 0.001). Figure 3B shows representative tumor perfusion images of mice treated on conventional dose continuous or high dose intermittent sorafenib at baseline, day 3, day7, and day10 after treatment. Thus, the high dose intermittent regimen showed enhanced and prolonged decrease in tumor perfusion relative to the conventional dose continuous and intermittent schedule.

## Discussion

Sorafenib is a multi-kinase inhibitor that has activity in RCC and hepatocellular cancer. The activity of sorafenib in RCC is felt to be primarily due to its inhibition of VEGFR2 on tumor endothelium resulting in antiangiogeic effects. While sorafenib has activity in patients with RCC, the median PFS associated with sorafenib treatment appears to be less than that seen with more potent inhibitors of the VEGFR2 pathway [18-21]. Data from Amato et al, suggest that higher doses of sorafenib might produce enhanced anti-tumor responses, but such doses given continuously are not tolerable for most patients with advanced RCC [2]. We investigated increased sorafenib dose administered intermittently (days 1-3 with 4 days break) in murine RCC xenograft models given as a potential means of increasing the efficacy of treatment without significant increase in toxicity.

Our experiments show that the high dose intermittent regimen of sorafenib exhibited enhanced antitumor activity compared to the conventional lower dose continuous schedule of sorafenib. Both regimens delivered the same overall dose, but the intermittent schedule allowed for a higher dose to be administered initially, followed by a short treatment break. ASL MRI and IHC studies in these animals suggested that this enhanced antitumor effect was mediated by enhanced anti-angiogenic effects associated with the higher dose and more sustained anti-angiogenic activity with intermittent dosing relative to continuous dosing.

Two factors may contribute to the superiority of the high dose intermittent regimen. The higher dose led to a greater reduction of tumor vessels and tumor perfusion than the lower dose. However, the 4 day treatment-free period did not compromise the efficacy of the high dose intermittent regimen compared to high dose continuous therapy. Thus, the treatment break was not detrimental. During the 4 days off sorafenib, some tumor blood flow returned, as the perfusion at day 7 was higher than day 3 in the high dose intermittent arm. Then, at day 10, blood flow was again reduced after 3 more days of high dose sorafenib. This is consistent with our prior work which showed that tumors that develop resistance to sorafenib exhibited restored ability to respond to sorafenib when implanted into naïve hosts [22]. Further, our prior study showed that even a few days off sorafenib ("drug holiday") appeared to be sufficient to restore sensitivity of RCC xenografts to sorafenib as measured by induction of tumor necrosis. These observations are also supported by clinical data that suggests restored sensitivity to VEGFR TKI including the rechallenge with the same agent (sunitinib) following a drug holiday [23].

These data may have direct clinical relevance. Other VEGFR pathway inhibitors have demonstrated similar schedule issues. As recently reported, the EFFECT trial showed that sunitinib given at 50 mg/day for 4 weeks followed by a 2 week break was more active than sunitinib 37.5 mg daily with the higher dose intermittent regimen showing a 9.9 month PFS vs a 7.1 month PFS for the lower dose continuous regimen [11]. Furthermore, tivozanib is administered at 1.5 mg/kg qd for 3 weeks out of 4 and has produced at response rate 30% and a median PFS of 15 months in patients with clear cell histology who had undergone a nephrectomy [18]. A recent dose escalation study in RCC patients showed that increasing the dose of sorafenib while maintaining the continuous dosing schedule was not feasible due to toxicities. However, the patients in whom the dose could be escalated appeared to have greater clinical benefit with longer PFS and greater response rates [24]. Our data suggest that dose escalation in addition to schedule alteration may improve efficacy with less toxicity.

The concept that a higher dose may be more effective is also consistent with a study by Houk et al which showed that in patients treated with sunitinib, higher blood levels of the drug correlated with longer PFS and overall survival [3]. It is likely that the higher dose in our study achieved a higher blood level in the mice. The notion that higher drug levels may correlate with better efficacy is further supported by the finding that hypertension, thought to be an independent pharmacodynamic marker of VEGFR inhibition, correlated with response in a retrospective study of patients treated with first or second-line sunitinib [4,5]. Patients with systolic BP >/=140 had a 12.5 month PFS and patients with no systolic hypertension had only a 2.5 month PFS.

Thus, as with other VEGFR pathway inhibitors, sorafenib's overall effect may be enhanced at higher doses, likely leading to higher blood levels, and more effective inhibition of VEGFR. This results in improved antiangiogenic activity and improved efficacy. The finding that the high dose showed similar activity at either an intermittent or continuous schedule would support the practice of allowing patients a break from therapy to recover from toxicities. While this data requires validation, it suggests that the therapeutic index for VEGFR TKI therapy of RCC may be maximal with higher dose intermittent therapy. Perhaps using this approach might optimize the impact of not just sorafenib and sunitinib, but other VEGFR inhibitors in RCC and possibly in other tumors that are responsive to VEGF targeted therapy.

## Conclusions

Sorafenib shows improved activity in RCC when administered as a high dose intermittent regimen. The mechanism by which this occurs appears to be improved antiangiogenic activity. Considerations of dosing and schedule of antiangiogenic agents may improve their therapeutic function.

## List of abbreviations

RCC: renal cell carcinoma; VEGF: vascular endothelial growth factor; TKI: tyrosine kinase inhibitor; ASL MRI: arterial spin labeled magnetic resonance imaging; CD: conventional dose; HD: high dose.

## Competing interests

Dr. Alsop is an inventor on patents related to the ASL MRI technique used in this study and receives institutional royalties for these patents from GE Healthcare, an MRI scanner vendor.

## Authors' contributions

The conception and design of the study was by RB, SNG, MA, and XW. XW, LZ and RB are responsible for acquisition and analysis of data and interpretation of data. MB and XW performed statistical analysis. VB and SS are responsible for the immunohistochemical analyses. The manuscript was drafted by RB and XW, and critically revised by RB, XW, SNG, DA, and MA. All authors read and approved the final manuscript.
